# Clinical Impact of Stool Polymerase Chain Reaction (PCR) Testing in Hospitalized Patients with Acute Diarrhea: A Retrospective Observational Study

**DOI:** 10.3390/biomedicines13051155

**Published:** 2025-05-09

**Authors:** Crina Fofiu, Daniela Dobru, Adina Andone, Victoria Ancuța Nyulas, Alina Boeriu

**Affiliations:** 1 Gstroenterology Department, University of Medicine Pharmacy, Sciences, and Technology “George Emil Palade” Targu Mures, 540142 Targu Mures, Romania; crina.fofiu@umfst.ro (C.F.); daniela.dobru@umfst.ro (D.D.); alina.boeriu@umfst.ro (A.B.); 2 Internal Medicine Department, Bistrița Clinical County Emergency Hospital, 420094 Bistrița, Romania; 3 Gastroenterology Department, Mures County Clinical Hospital, 540103 Targu Mures, Romania; 4 Department of Medical Informatics and Biostatistics, University of Medicine Pharmacy, Sciences, and Technology “George Emil Palade” Targu Mures, 540142 Targu Mures, Romania; victoria.rus@umfst.ro

**Keywords:** stool PCR, acute diarrheal illnesses, *Campylobacter* spp., *Clostridioides difficile*

## Abstract

**Background/Objectives**: Acute diarrheal illnesses are a major cause of hospital admissions, particularly in immunocompromised patients. Traditional diagnostic methods are slow and often insensitive, delaying treatment. In contrast, PCR panels provide rapid, sensitive detection of multiple pathogens. This study evaluates stool PCR testing in hospitalized adults and its impact on clinical decisions and antimicrobial stewardship. **Methods**: We conducted a retrospective study at Bistrița County Hospital, Romania (September 2023–September 2024), including 75 adults with acute diarrhea and negative conventional stool tests. PCR testing (VIASURE panels I and II) detected 11 bacteria, 6 viruses, and 5 parasites. Clinical and therapeutic data were analyzed, and logistic regression identified predictors of PCR positivity and adverse outcomes. **Results**: PCR was positive in 78% of cases, with *Campylobacter* spp. (57.6%) and *Clostridioides difficile* (20.3%) being the most common. Bloody diarrhea independently predicted PCR positivity (OR 9.78, *p* = 0.047). Immunosuppression and end-stage liver disease were linked to worse outcomes. PCR results led to antimicrobial therapy adjustments in 40 patients (*p* = 0.001), correcting inappropriate antibiotic use in 66% of those receiving empirical treatment. Targeted therapy significantly reduced antimicrobial duration from 7 to 5 days (*p* = 0.00001). **Conclusions**: Stool PCR testing enhances pathogen detection, guides targeted therapy, and reduces inappropriate antibiotic use, supporting antimicrobial stewardship and improving outcomes in selected hospitalized patients.

## 1. Introduction

Acute diarrheal infection is a common cause of outpatient visits, hospitalizations, and diminished quality of life, occurring both in domestic settings and among international travelers. Globally, it is responsible for over one million deaths annually [1]. Diarrheal diseases rank among the leading causes of mortality in children under five years of age, particularly in low- and middle-income countries [2]. In contrast, the burden of diarrheal illness in adult populations has not been as comprehensively studied. Although episodes in adults are often milder, the overall impact on healthcare systems, economic productivity, and public health remains substantial [3]. Therefore, evaluating the economic burden of diarrheal disease in the adult population is essential to identify potential cost-saving strategies and inform policy decisions aimed at improving health outcomes and quality of life.

Infectious diarrhea is caused by a variety of enteric pathogens, including bacteria, parasites, viruses, and, less commonly, fungi. The most frequently implicated bacterial pathogens include *Escherichia coli* (*E. coli*), *Campylobacter* spp., *Salmonella* spp., *Shigella* spp., *Yersinia enterocolitica*, and *Clostridioides difficile* (*C. difficile*) [4,5].

Microbiological investigation is generally not necessary for immunocompetent patients with acute, self-limited, or viral diarrheal illness, as approximately half of such cases resolve within 24 h of onset. However, in hospitalized patients with persistent symptoms, pathogen-directed therapy based on accurate microbial identification is essential.

Stool testing—including culture and microscopic examination—is typically the initial diagnostic approach in cases of diarrhea associated with fever, bloody or mucoid stools, severe abdominal pain, or clinical signs of sepsis [5]. Nevertheless, these conventional methods are time-consuming and often lack the sensitivity and specificity required for reliable diagnosis.

An incubation period of up to three days is often required for bacterial pathogens to reach detectable levels before colonies can be identified through traditional culture methods, potentially resulting in significant delays in diagnosis and the initiation of appropriate treatment. Furthermore, certain bacterial pathogens, such as *Campylobacter* spp., have specific and demanding culture requirements, making them difficult to isolate and resulting in reduced sensitivity, which may contribute to underdiagnosis [6]. As an alternative, polymerase chain reaction (PCR)-based stool testing has been developed. This method allows for the rapid and accurate detection of multiple pathogens simultaneously from a single sample, significantly reducing the time to diagnosis to just a few hours while improving laboratory efficiency [7,8]. However, the widespread implementation of PCR panels introduces new challenges. Clinicians may lack familiarity with interpreting test results or may overutilize diagnostic tests in the absence of clear clinical indications. Therefore, diagnostic stewardship is essential to ensure the appropriate and effective use of molecular diagnostic tools.

This study aimed to evaluate the appropriateness of stool PCR panel testing in hospitalized patients and its impact on patient care. The objective was to determine whether the use of this test can inform local recommendations for diagnostic and therapeutic stewardship strategies.

## 2. Materials and Methods

### 2.1. Study Population

This retrospective observational study was conducted at Bistrița County Hospital, an emergency care center located in Bistrița, Bistrița-Năsăud, Romania, over a one-year period from September 2023 to September 2024. Data were collected from the medical records of patients admitted to various departments, including Internal Medicine, Infectious Diseases, and Surgery. Eligible participants included hospitalized patients diagnosed with acute diarrhea at the time of admission, defined as the passage of three or more loose or watery stools per day with symptom onset within the preceding 14 days. Exclusion criteria included patients under 18 years of age, individuals with a diagnosis of chronic diarrhea, and pregnant or lactating women. The study protocol was approved by the Institutional Ethics Committee of Bistrița County Hospital (Approval No. 3690/2/25.01.2025).

Data collected included demographic characteristics, clinical presentation, comorbidities, recent antibiotic exposure, recent use of corticosteroids or immunosuppressive agents, recent surgical interventions, and immunosuppressive conditions such as malignancy, autoimmune diseases, inflammatory bowel diseases, and cancer. Additional parameters assessed were duration of hospital stay, laboratory findings, results of conventional bacterial stool tests, stool PCR panel findings, appropriateness of antibiotic prescribing, duration of antibiotic treatment, and the need for therapy modification based on PCR results.

Optimal antibiotic therapy was defined as the use of agents proven to effectively target the identified bacterial pathogen, with appropriate selection, dosing, and duration in accordance with established clinical guidelines and professional standards of care. Antibiotic use was considered inappropriate when empirical therapy was initiated in patients subsequently diagnosed via stool PCR with specific pathogens such as *Clostridioides difficile*, *Campylobacter* spp., or *Shigella* spp., where targeted or alternative management may be indicated. Inappropriate use was also defined as the prescription of antibiotics in cases with negative PCR results or detection of viral pathogens, where antibiotic therapy is not indicated.

### 2.2. Outcome Comparison and Conventional Diagnostic Testing

We compared disease outcomes between patients with positive stool PCR panel results and those with negative findings, as well as the impact of PCR results on therapeutic decisions in both groups. The following were considered adverse outcomes: sepsis, septic shock, and in-hospital mortality.

All patients included in the study initially underwent conventional diagnostic testing for acute diarrhea. This included microscopic examination for ova and parasites, as well as bacterial stool cultures. In addition, testing for *Clostridioides difficile* infection was performed. Our institution’s standard stool culture protocol focuses on the detection of Salmonella spp. and Shigella spp., using the VIATEK automated system (bioMérieux, Marcy-l’Étoile, France). For *C. difficile* assessment, a two-step diagnostic approach was employed, consisting of glutamate dehydrogenase (GDH) screening followed by enzyme immunoassay (EIA) for toxin A and B detection (SD Biosensor, Inc., Suwon, Republic of Korea).

### 2.3. Stool Multiplex PCR Assay

Stool PCR analysis was performed using the DT Prime Real-Time PCR system (DNA-Technology, Moscow, Russia). Total nucleic acids were extracted from stool specimens using the VIASURE extraction kit (Certest Biotec, Zaragoza, Spain), in accordance with the manufacturer’s instructions. Two gastrointestinal panels from the VIASURE PCR kit were employed: Gastrointestinal Panel I and Gastrointestinal Panel II. Together, these panels allow for the detection of 11 bacterial, 6 viral, and 5 parasitic pathogens. Gastrointestinal Panel I primarily detects viral pathogens, including norovirus GI and GII, rotavirus, adenovirus, astrovirus, and sapovirus. It also identifies selected bacterial pathogens such as *Salmonella* spp., *Campylobacter* spp., *Shigella* spp., and *Yersinia enterocolitica*, as well as parasitic organisms including *Cryptosporidium* spp., *Entamoeba histolytica*, and *Giardia lamblia*. Gastrointestinal Panel II is designed for the specific identification and differentiation of various bacterial as well as parasitic pathogens. These include *Salmonella* spp., *Campylobacter* spp., *Shigella* spp., *Aeromonas* spp., *enterohemorrhagic Escherichia coli* (*EHEC*), *enteropathogenic E. coli* (*EPEC*), *enteroinvasive E. coli* (*EIEC*), *Shiga toxin-producing E. coli* (*STEC*), *Clostridioides difficile* (including detection of toxin B), and *Yersinia enterocolitica*. Parasitic pathogens included in this panel are *Blastocystis hominis*, *Dientamoeba fragilis*, *Entamoeba histolytica*, and *Cryptosporidium* spp. Quality control was ensured through the use of internal positive and negative controls included in each PCR run. All assays were performed according to the manufacturer’s validated protocols, and laboratory personnel were blinded to clinical outcomes to avoid bias. Additionally, routine calibration of equipment and periodic proficiency testing were conducted to maintain the reliability and reproducibility of the results. Reported sensitivity ranges between 95% and 100%, and specificity between 96% and 100%, depending on the individual pathogen

### 2.4. Statistical Analysis

Statistical analyses were performed using SPSS Statistics for Windows, version 20.0 (SPSS Inc., Chicago, IL, USA). Categorical variables were analyzed using the Chi-square test or Fisher’s exact test, as appropriate, to evaluate associations and differences between groups. The Kolmogorov–Smirnov test was applied to assess the normality of distribution for continuous variables. For comparison of continuous variables between groups, Student’s *t*-test was used for normally distributed data. To identify independent predictors of unfavorable outcomes among patients with positive stool PCR results, univariate and multivariate logistic regression analyses were conducted. Odds ratios (ORs) and 95% confidence intervals (CIs) were calculated. A *p*-value ≤ 0.05 was considered statistically significant.

## 3. Results

### 3.1. Baseline Characteristics

A total of 75 patients were hospitalized with acute diarrhea over a one-year period, from September 2023 to September 2024. All patients initially tested negative using conventional diagnostic methods, including bacterial culture and microscopy. Based on clinical presentation and laboratory findings, stool polymerase chain reaction (PCR) testing was subsequently performed. Baseline and clinical characteristics are summarized in Table 1. The mean patient age was 60.4 years, with a nearly equal gender distribution (38 females and 37 males). The majority of patients were admitted to the Department of Internal Medicine (64%), followed by the Department of Infectious Diseases (22.6%), and the Department of Surgery (13.4%). The mean length of hospital stay was 10 days, with a maximum duration of 39 days.

When analyzing potential risk factors for enteric infections—including comorbidities, immunosuppression, recent surgery (within 30 days), prior antibiotic exposure (<30 days), and immunosuppressive therapy—we found that cardiovascular disease and immunosuppression were the most common comorbid conditions in the PCR-positive group, present in 48% and 45.3% of cases, respectively. However, these associations did not reach statistical significance. Recent antibiotic exposure and ongoing immunosuppressive therapy were more frequently observed in the PCR-positive group (Table 1).

### 3.2. Bacterial Pathogens Detected by Stool PCR

**Figure 1:**
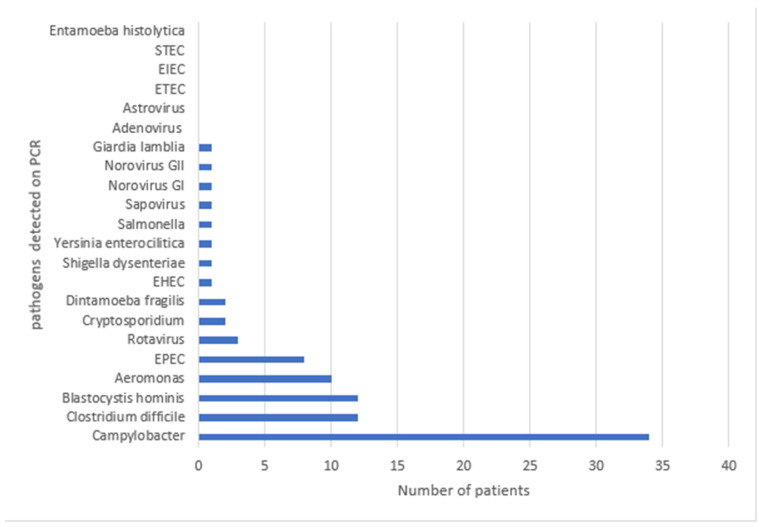
Pathogens detected on stool PCR testing.

From the total of 75 GI panels, 59 yielded positive results (78%) (Figure 1).

The most frequently identified pathogen was *Campylobacter* spp., detected in 34 patients (57.6%), followed by *Clostridioides difficile* and *Blastocystis hominis*, each found in 12 patients (20.3%), and *Aeromonas* in 10 patients (16.9%). In 28 out of 59 PCR-positive cases (47.5%), more than one pathogen was identified. The highest number of pathogens detected in a single sample was three, with combinations including *Blastocystis hominis*, *Campylobacter* spp., and *Cryptosporidium* spp., or *Aeromonas*, *Campylobacter* spp., and *enteropathogenic Escherichia coli* (*EPEC*). Pathogens not detected by the panel included *enteroinvasive E. coli* (*EIEC*), *enterotoxigenic E. coli* (*ETEC*), *Entamoeba histolytica*, adenovirus, and astrovirus (Figure 1).

In total, the stool PCR assay identified 91 pathogens: 6 viruses (6.6%)—including 3 rotavirus, 1 sapovirus, 1 norovirus GI, and 1 norovirus GII; 17 parasites (18.7%)—consisting of 12 *Blastocystis hominis*, 2 *Cryptosporidium* spp., 2 *Dientamoeba fragilis*, and 1 *Giardia lamblia*; and 68 bacteria (74.7%)—including 34 *Campylobacter* spp., 12 *C. difficile*, 10 *Aeromonas*, 8 EPEC, 1 enterohemorrhagic *E. coli* (EHEC), 1 *Salmonella* spp., 1 *Shigella dysenteriae*, and 1 *Yersinia enterocolitica*.

Among the 28 patients with multiple pathogens detected, the most frequent co-infection was *Blastocystis hominis* with *Campylobacter* spp., found in 9 cases (32.1%). Of the 12 patients who tested positive for *C. difficile* by PCR, 5 (41.7%) had confirmatory enzyme immunoassay results for toxins A and B.

### 3.3. Comparison Characteristics Based on the Positivity of Stool PCR Testing

We analyzed the association between stool PCR positivity and the hospital department to which patients were admitted. The majority of PCR-positive cases (39 out of 59; 66.1%) were admitted to the Department of Internal Medicine, a finding that reached statistical significance (*p* = 0.04). In terms of clinical presentation, loose stools were reported in 58% of patients, while watery and bloody diarrhea were noted in 25% of cases (Table 1). Additionally, 74% of patients experienced abdominal pain. When comparing clinical symptoms between patients with positive and negative stool PCR results, bloody diarrhea was significantly more frequent among PCR-positive individuals (*p* = 0.04).

Regarding exposure to medications associated with an increased risk of gastrointestinal infections. Among PCR-positive patients, 13.6% had recent antibiotic exposure, 3.4% had received corticosteroids, and 10.2% were on immunosuppressive therapy. However, no statistically significant associations were observed between stool PCR positivity and prior exposure to antibiotics (*p* = 0.69), steroids (*p* = 0.51), or immunosuppressants (*p* = 0.39).

We analyzed whether the presence of comorbidities or an immunocompromised state was associated with an increased likelihood of detecting multiple pathogens via stool PCR. Among the 28 patients with more than one pathogen identified, 16 (57.1%) had underlying comorbidities. In comparison, 20 out of 31 patients (64.5%) with a single pathogen also had comorbid conditions. This difference was not statistically significant (*p* = 0.562), indicating no meaningful correlation between the presence of comorbidities and the detection of multiple pathogens.

### 3.4. Clinical Factors That Supported Detection of Bacterial Pathogens by Stool PCR

To identify factors predictive of stool PCR positivity, a univariate logistic regression analysis was conducted. Comorbidities—including cardiovascular disease, immunosuppression conditions, diabetes mellitus, end-stage liver disease, recent surgery, and malignancy—as well as laboratory blood test results, were not significantly associated with PCR positivity. The only independent predictor of enteric pathogen detection by stool PCR was the presence of bloody diarrhea (odds ratio [OR] 9.78; 95% confidence interval [CI]: 1.03–92.21; *p* = 0.047) (Table 2).

### 3.5. Risk Factors for Adverse Outcomes Among Patients with Positive Stool PCR Results

A multivariate logistic regression analysis was performed to identify risk factors associated with adverse outcomes among patients with positive stool PCR results (Table 3). Variables included in the model were comorbidities, diarrhea-related symptoms, type of gastrointestinal infection, and laboratory parameters. Adverse outcomes were defined as the development of sepsis, septic shock, or death.

The presence of end-stage liver disease was significantly associated with increased risk for all adverse outcomes: death (OR 7.79; 95% CI: 1.06–57.19; *p* = 0.043), sepsis (OR 77.65; 95% CI: 4.12–1462.13; *p* = 0.004), and septic shock (OR 8.72; 95% CI: 1.16–65.10; *p* = 0.035). Immunocompromised status was associated with an elevated risk of death (OR 5.09; 95% CI: 0.81–31.89; *p* = 0.048). Additionally, the risk of sepsis was significantly higher in patients with infectious diarrhea who presented abdominal pain (OR 10.8; 95% CI: 1.30–89.34; *p* = 0.027).

Based on pathogen-specific analysis, infections with *Campylobacter* spp. and *Clostridioides difficile* were significantly associated with a higher risk of sepsis (OR 7.48; 95% CI: 0.77–72.31; *p* = 0.044 and OR 13.89; 95% CI: 2.32–83.04; *p* = 0.004, respectively). Other variables, including elevated inflammatory markers, cardiovascular comorbidities, diabetes, recent surgery, and immunosuppression, were not found to be independent predictors of severe outcomes in the PCR-positive patient group (Table 3).

### 3.6. Antibiotic Stewardship

Empirical antibiotic therapy was initiated in 53 patients (70.7%) after sample collection, predominantly with broad-spectrum agents: fluoroquinolones in 23 patients (43.4%), metronidazole in 18 patients (34.0%), and cephalosporins in 12 patients (22.6%). Using a chi-square test, we compared the initiation of empirical antibiotic therapy between PCR-positive and PCR-negative groups. Based on clinical judgment and severity of presentation—including symptoms such as abdominal pain and bloody diarrhea, as well as abnormal laboratory findings (leukocytosis, elevated C-reactive protein, and procalcitonin)—empirical antimicrobial therapy was started in 50 out of 59 patients (84.7%) in the PCR-positive group, compared to 3 out of 16 patients (18.8%) in the PCR-negative group (*p* = 0.001).

**Figure 2:**
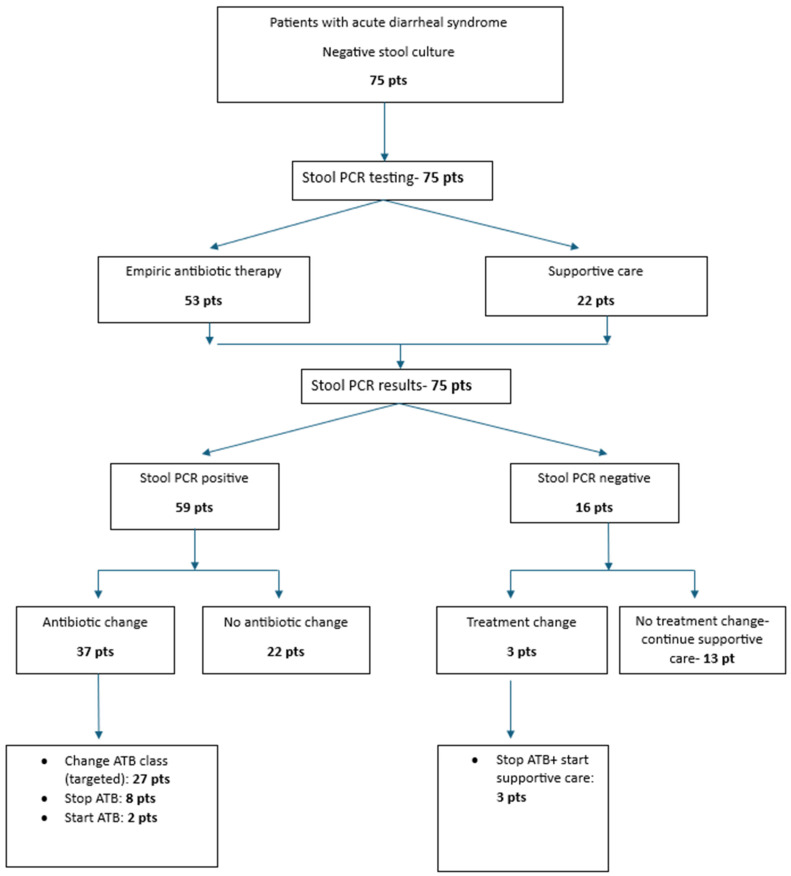
Patients’characteristics and antibiotic stewardship.

Following the receipt of stool PCR results, antimicrobial therapy was adjusted in 40 patients: 37 from the PCR-positive group and 3 from the PCR-negative group. Modifications included initiation of targeted antibiotics, discontinuation of empirical therapy, or switching to a more appropriate antibiotic class (Figure 2).

Among the 53 patients who received empirical antibiotics, inappropriate use was identified in 35 cases (66.0%) after PCR results became available. In 27 of these cases, therapy did not appropriately target the detected pathogen. The most frequent prescribing error involved the use of metronidazole or fluoroquinolones for *Campylobacter* spp. infections (21/35 cases; 60%), followed by the use of fluoroquinolones for *C. difficile* infections (6/35 cases; 17.1%). Additionally, in 8 out of 35 cases (22.9%), antibiotics were unnecessarily administered to patients with viral pathogens (e.g., norovirus, sapovirus) or parasitic infections (e.g., *Dientamoeba fragilis*).

To assess whether stool PCR results influenced antibiotic prescribing practices, we evaluated the correlation between PCR findings and subsequent treatment modifications. A significant association was identified between positive stool PCR results and changes in antimicrobial therapy, with 40 out of 59 PCR-positive patients (67.8%) requiring adjustments based on the identified pathogen (*p* = 0.001).

We also analyzed the duration of antimicrobial therapy in relation to stool PCR findings. In most cases, the mean duration of antimicrobial treatment decreased from 7 days to 5 days when therapy was adjusted following PCR results (*p* = 0.00001). Among 35 patients who initially received inappropriate empirical antibiotics, 25 (71.4%) had their 7-day regimen of metronidazole or fluoroquinolones switched to a 5-day course of azithromycin after *Campylobacter* spp. was identified. Conversely, targeted therapy for *Clostridioides difficile* infections often requires extended treatment durations, with some cases necessitating up to 10 days of appropriate antimicrobial therapy.

## 4. Discussion

While diarrheal mortality has significantly declined in young children due to public health measures such as widespread rotavirus immunization, improved sanitation, and hygiene practices, mortality in adults—particularly older individuals with comorbid conditions—remains a persistent concern. In such populations, early assessment of diarrheal severity and prompt identification of the causative pathogen are essential for guiding appropriate therapy [3]. Traditional stool culture methods often require up to three days to yield results and may fail to identify certain bacterial species. As a result, more rapid molecular diagnostic tools, including stool PCR assays, have been developed and implemented in selected healthcare settings.

The objective of our study was to evaluate the clinical utility of stool PCR testing in guiding therapeutic decision-making for patients presenting with acute diarrheal syndrome. Specifically, we aimed to assess the impact of pathogen-specific PCR results on the initiation and modification of targeted antimicrobial therapy.

Consistent with findings from previous studies, our research supports the utility of PCR panels in improving the detection of enteric pathogens that are often missed by conventional diagnostic methods. This enhanced diagnostic capability was shown to impact clinical decision-making, leading to more appropriate antibiotic use and reinforcing the principles of antimicrobial stewardship [9,10,11,12,13].

In our study population of 75 patients presenting with acute diarrheal syndrome and negative results on conventional stool testing, 78% were found to have a positive result on stool PCR. This high positivity rate reflects the rigorous clinical selection criteria used to determine which patients warranted advanced diagnostic testing. Ultimately, the combination of clinical presentation and supportive laboratory parameters proved useful in guiding treatment decisions in PCR-positive cases.

A review of the literature reveals considerable variability in PCR positivity rates, ranging from 30% to 70% depending on patient population and testing criteria [14,15]. Although current guidelines generally discourage routine stool PCR testing for all patients with acute diarrhea—primarily due to cost considerations—several studies have shown that patients undergoing gastrointestinal (GI) PCR testing are less likely to require invasive diagnostic procedures, such as endoscopic or radiologic evaluations [16,17]. Additionally, GI PCR testing has been associated with reduced antibiotic use and decreased need for isolation precautions [17,18]. These findings suggest that when used appropriately—guided by careful clinical judgment—stool PCR testing may contribute to both improved patient management and cost savings in routine clinical practice.

Given the high sensitivity of PCR, which enables detection of both viable and non-viable organisms, careful patient selection for testing is essential. In our study, we sought to identify clinical and laboratory criteria to better define the subgroup of patients at increased risk for enteric infections. Factors such as the type of diarrhea, associated symptoms (e.g., abdominal pain, bloody stools), altered laboratory values (e.g., leukocytosis, elevated CRP), presence of comorbidities, and immunological status were analyzed to guide appropriate test utilization. Our analysis identified the presence of blood in the stool as an independent predictor of infectious diarrhea, observed in 30.5% of PCR-positive patients compared to 6.2% of PCR-negative patients (*p* = 0.0047). These findings are consistent with those of a large retrospective study by Kwack et al., which included 725 patients with acute diarrheal syndrome. In that study, bloody diarrhea was also significantly more common in PCR-positive patients than in PCR-negative ones (22.6% vs. 14.8%; *p* = 0.01) [19]. These results support the use of bloody diarrhea as a key clinical indicator for considering stool PCR testing in patients with acute diarrhea and a negative conventional microbiological workup.

Other laboratory parameters—such as leukocytosis, elevated C-reactive protein, procalcitonin, and hypoalbuminemia—did not show a statistically significant correlation with PCR positivity in our cohort. Furthermore, we were unable to identify additional predictive factors, including underlying comorbidities or immunosuppressive states, that were significantly associated with positive PCR results.

Regarding the impact of enteric infections on patient outcomes, our study demonstrated that immunocompromised individuals are at increased risk for adverse outcomes, and a more severe disease course should be anticipated in this population when infectious diarrhea is present. In particular, patients with end-stage liver disease (cirrhosis) in the PCR-positive group exhibited the highest risk of developing sepsis, septic shock, and death (*p* = 0.004, *p* = 0.03, and *p* = 0.03, respectively). These findings are supported by recent evidence. Xu et al., in a systematic review, examined the role of bacterial infections in the clinical progression of acute-on-chronic liver failure, while Pisipati et al. specifically addressed *Campylobacter* spp. bacteremia in patients with cirrhosis. Their findings underscore the importance of early and appropriate antimicrobial therapy, particularly in the context of rising antimicrobial resistance among cirrhotic patients [20,21,22]. Collectively, these studies emphasize the need for early pathogen identification and risk stratification in patients with acute diarrheal syndrome, especially those with underlying liver disease or immunocompromised status.

In our cohort, the second most commonly detected pathogen on stool PCR was *Clostridioides difficile* (20.3%), identified in 12 of 59 PCR-positive patients. These findings may reflect an increasing prevalence of community-acquired *C. difficile* infection and potentially greater virulence. This trend is likely influenced by the widespread use of antibiotics, which disrupt the gut microbiota and increase susceptibility to *C. difficile* colonization and infection. Notably, 13.6% of PCR-positive patients in our study had recent antibiotic exposure. However, no statistically significant association was observed between prior antibiotic use and the onset of diarrhea in this group (*p* = 0.69). These findings highlight the ongoing importance of antimicrobial stewardship not only in the prevention of *C. difficile* infection but also in the broader management of infectious diarrheal illnesses.

Our findings regarding the role of stool PCR testing for *Clostridioides difficile* infection (CDI) are consistent with existing literature. Among the 12 patients who tested positive for C. difficile via PCR in our study, only 5 had a positive enzyme immunoassay (EIA) result for toxins A and B, demonstrating the higher sensitivity of PCR compared to EIA. While EIA is known to have limited sensitivity [23,24,25], nucleic acid amplification tests (NAATs), including PCR, may detect *C. difficile* DNA in individuals who are colonized but not clinically infected [26,27]. To address this diagnostic limitation, Infectious Diseases guidelines—including those from the European Society of Clinical Microbiology and Infectious Diseases (ESCMID)—recommend a two-step testing algorithm that combines EIA and PCR to improve diagnostic accuracy [28].

However, the adoption of this diagnostic algorithm has introduced a new clinical challenge: managing patients who test positive on PCR but negative on EIA (PCR+/EIA−). Recent studies have shown that these patients may still experience CDI-related complications comparable to those with confirmed toxin-positive disease [29,30,31]. ESCMID guidelines suggest that in cases of severe, persistent, or otherwise unexplained diarrhea with a clinical presentation strongly suggestive of CDI, treatment should still be considered, even in the absence of toxin detection [28]. In our study, all 12 patients with PCR-positive results for *C. difficile* exhibited clinical and laboratory features consistent with CDI, and all required targeted antibiotic therapy.

Additionally, in our cohort, more than one pathogen was detected by PCR in 28 patients (47.5%). We hypothesized that immunocompromised individuals or those with multiple comorbidities would be more prone to polymicrobial infections. However, no significant association was found between the presence of multiple pathogens and either immunosuppression or comorbidity burden (*p* = 0.562). This observation aligns with findings from Mannstadt et al., who conducted a large retrospective study and reported a lower prevalence of multiple pathogen detection (27%), with no significant correlation between polymicrobial infection and the presence of comorbidities [32].

Stewardship programs emphasize the importance of optimizing antibiotic use to improve patient outcomes and reduce resistance. Recent studies have demonstrated that the use of stool PCR testing can contribute to earlier initiation of appropriate antimicrobial therapy and shorter hospital stays when compared to traditional diagnostic methods [11,33,34,35,36]. In our study, 53 out of 75 patients (70.7%) received empirical antibiotic treatment, based on clinical and laboratory indicators of disease severity. The decision to initiate empirical therapy was significantly more frequent in the PCR-positive group (50/59 patients) compared to the PCR-negative group (3/16 patients), suggesting that clinicians are more likely to treat aggressively when clinical suspicion of infection is high. This pattern is commonly observed in clinical practice, especially in patients with underlying comorbidities. Similar findings were reported by Kwack et al. in a large retrospective study, which also demonstrated a positive correlation between empirical antibiotic initiation based on clinical judgment and subsequent positivity of stool multiplex PCR results [19].

Despite the value of early empirical therapy, inappropriate antibiotic use remains a concern. In our study, inappropriate antibiotic therapy was documented in 35 patients (66%) following PCR testing, leading to discontinuation or adjustment of the prescribed antimicrobial regimen. Specifically, 27 patients received antibiotics that did not target the identified pathogen—most commonly metronidazole or ciprofloxacin for *Campylobacter* spp., and fluoroquinolones or cephalosporins for C. difficile. Additionally, in 8 patients who were empirically treated with antibiotics, a viral or parasitic etiology of diarrhea was confirmed by PCR.

These findings are consistent with those of Cybulski et al., who, in a large prospective study, compared stool PCR testing with conventional stool culture. They found that patients tested with PCR were more likely to receive targeted rather than empirical therapy, had a shorter median time from sample collection to the initiation of appropriate antimicrobial treatment, and were more likely to have empirical therapy discontinued when deemed unnecessary [13]. Collectively, these data support the role of PCR testing as a valuable tool in antimicrobial stewardship by improving diagnostic accuracy and promoting more appropriate treatment decisions.

With regard to empirical antibiotic treatment for *Campylobacter* infections, most clinical guidelines recommend either a fluoroquinolone (such as ciprofloxacin) or a macrolide (such as azithromycin), with the choice depending on regional resistance patterns. Given the rising global resistance to fluoroquinolones—ranging from 30% to over 70%—azithromycin has become the preferred first-line agent for *Campylobacter* gastroenteritis in many settings [37,38]. In accordance with local epidemiological data and antimicrobial resistance trends, our hospital protocol recommends azithromycin as the initial treatment of choice for *Campylobacter* infections. As such, the empirical use of fluoroquinolones in patients subsequently diagnosed with *Campylobacter* via PCR was considered inappropriate in our analysis. In these cases, therapy was appropriately adjusted by switching from fluoroquinolones to macrolides.

In addition, antimicrobial therapy was discontinued in eight patients whose PCR results identified viral or parasitic pathogens. These individuals were managed with supportive care only, in line with local treatment guidelines. This targeted approach helped prevent unnecessary antibiotic use and supports broader efforts to combat antimicrobial resistance.

When analyzing the impact of PCR testing on clinical management, we identified a strong correlation between PCR results and subsequent treatment adjustments. A total of 40 patients across both PCR-positive and PCR-negative groups required changes to their treatment regimen based on pathogen identification (*p* = 0.001). Specifically, PCR-guided decision-making led to the discontinuation of empirical antibiotics in 35 cases and the initiation of targeted antimicrobial therapy in 29 cases. These interventions enhanced the appropriateness of care and have the potential to positively impact patient outcomes.

Our study provides evidence that the use of stool PCR testing can contribute to a reduction in the duration of antibiotic therapy. Following identification of the causative pathogen and initiation of targeted therapy, the duration of antimicrobial treatment was significantly reduced—from 7 to 5 days—in 71.4% of cases (*p* = 0.00001). This reduction has important implications for decreasing hospital length of stay and reducing overall healthcare costs. Supporting this, a comprehensive review by Freeman et al., conducted on behalf of the UK National Health Service, evaluated the economic impact of using multiplex pathogen panels (MPPs) in the diagnosis of acute gastroenteritis (AGE). Their economic model estimated that the use of MPPs could result in up to a 50% reduction in hospital stay duration, ultimately offsetting the higher initial cost of molecular testing through downstream savings [39,40].

Future research comparing patient outcomes and resource utilization before and after the implementation of PCR testing could provide a comprehensive cost–benefit analysis. Such data could support a shift in clinical practice—from empiric to targeted therapy—and reinforce the role of molecular diagnostics as a cornerstone of antimicrobial stewardship in acute diarrheal disease management.

### Study Limitations

Our study has several limitations. A larger sample size would likely yield a higher proportion of PCR-negative results, which would allow for a more accurate evaluation of risk factors associated with enteric infections and improve assessment of testing appropriateness across different patient populations. Additionally, some pathogens may not have been detected by the conventional diagnostic methods used in our institution, which could explain the greater reliance on molecular testing. Studies that incorporated specialized culture techniques—such as those for *Campylobacter*, *Vibrio*, and *Yersinia*—have reported higher diagnostic yields and better agreement with molecular methods. Incorporating additional antigen-based tests could also enhance diagnostic sensitivity [11].

Finally, our study did not yield information on genes associated with antibiotic resistance, despite the potential of real-time PCR testing to provide such data. Antibiotic resistance is a significant concern in healthcare, as identifying whether a bacterial strain is resistant to specific antibiotics can guide treatment decisions. However, the testing of antibiotic resistance genes would require additional steps and analysis, leading to increased resource allocation and overall testing costs. Currently, our hospital protocol dictates the continuation of antibiogram testing following a positive stool culture, and in cases of positive stool PCR results, antibiotics are prescribed based on local antibiotic resistance patterns.

## 5. Conclusions

Our study highlights the role of stool PCR testing in optimizing antimicrobial therapy. Stool PCR panels represent a significant advancement in the ability to diagnose viral gastroenteritis and will likely transform our understanding of the epidemiology of this clinical syndrome. We demonstrated the value of PCR testing in guiding targeted antibiotic therapy and reducing empirical antimicrobial use when appropriately indicated. Overall, the use of stool PCR testing in the management of acute diarrhea aligns with the objectives of antimicrobial stewardship by promoting judicious antibiotic use, enhancing clinical outcomes through targeted therapy, and supporting broader public health efforts.

## Figures and Tables

**Table 1:** Baseline and clinical characteristics.

Variable	Total 75	Negative PCR 16	Positive PCR 59	*p* Value
Age, years, mean ± SD	60.4 ± 18.1	59.19 ± 18	60.45 ± 18	
Sex				0.23
Male, *n* (%)	37 (49.3)	10 (62.5)	27 (45.8)	
Female, *n* (%)	38 (50.7)	6 (37.5)	32 (54.2)	
Department				0.04
Internal medicine, *n* (%)	48 (64)	9 (56.2)	39 (66.1)	
Infectious diseases, *n* (%)	17 (22.6)	2 (12.5)	15 (24.4)	
Surgery Dep, *n* (%)	10 (13.4)	5 (31.1)	5 (8.5)	
Symptoms:				
Loose stools, *n* (%)	44 (58.7)	8 (39)	36 (61)	0.42
Watery diarrhea, *n* (%)	19 (25.3)	5 (31.2)	14 (23.7)	0.54
Bloody diarrhea, *n* (%)	19 (25.3)	1 (6.2)	18 (30.5)	0.04
Abdominal pain, *n* (%)	56 (74.7)	13 (81.2)	43 (72.9)	0.74
Comorbidities				
Cardiovascular, *n* (%)	36 (48)	7 (43.8)	29 (49.2)	0.70
DM, *n* (%)	15 (20)	4 (25)	11 (18.6)	0.72
Nephropathy, *n* (%)	23 (30.7)	6 (37.5)	17 (28.8)	0.50
End-stage liv dis, *n* (%)	21 (28)	5 (31.2)	16 (27.1)	0.74
Surgery < 1 month, *n* (%)	17 (22.7)	5 (31.2)	12 (20.3)	0.35
Cancer, *n* (%)	13 (17.3)	4 (25)	9 (15.3)	0.45
Immunosuppression, *n* (%)	34 (45.3)	9 (56.2)	25 (42.4)	0.32
High-Risk medication				
ATB, *n* (%)	11 (14.7)	3 (18.8)	8 (13.6)	0.69
Corticosteroids, *n* (%)	3 (4)	1 (6.2)	2 (3.4)	0.51
Immunosuppressants, *n* (%)	9 (12)	3 (18.8)	6 (10.2)	0.39

**Table 2:** Variables associated with stool PCR positivity.

		Positive PCR	
	OR	95% CI	*p* Value
Symptoms			
Watery diarrhea	0.52	0.08–3.32	0.49
Loose stools	1.26	0.24–6.43	0.78
Bloody diarrhea	9.78	1.03–92.91	0.047
Abdominal pain	0.63	0.13–2.96	0.65
Comorbidities			
DM	0.69	0.17–2.79	0.61
CV disease	0.63	0.27–5.61	0.77
End-stage liv dis	0.70	0.19–2.61	0.60
Recent surgery	0.59	0.13–2.63	0.49
Cancer	0.51	0.07–3.48	0.49
Immunosuppression	1.05	0.24–4.58	0.94
Lab tests			
WBC > 15.000	1.00	1.00–1.00	0.16
CRP > 5 mg/dL	1.00	1.00–1.00	0.94
PCT > 1 ng/mL	0.82	0.65–1.40	0.95
Albumin < 3 g/L	0.36	0.09–1.34	0.12
Recent ATB	0.41	0.06–2.70	0.35

**Table 3:** Multivariate analysis of risk factors for poor prognosis in the positive PCR testing group.

		Sepsis			Septic Shock			Death	
Covariate	OR	95% CI	*p* Value	OR	95% CI	*p* Value	OR	95% CI	*p* Value
Symptoms									
Watery diarrhea	0.97	0.05–16.6	0.98	0.28	0.01–5.15	0.39			
Loose stools	0.39	0.03–4.73	0.46	0.44	0.05–3.54	0.44			
Bloody diarrhea	0.41	0.02–6.06	0.52	0.17	0.01–2.57	0.20			
Abdominal pain	10.8	1.30–89.34	0.02	0.00	-	0.99			
Comorbidities									
DM	0.20	0.00–25.68	0.51	0.00	-	0.99	0.00	-	0.99
CV disease	3.49	0.29–41.45	0.32	0.94	0.09–9.24	0.96	0.00	-	0.99
End-stage liv dis	77.65	4.12–1462.1	0.004	8.72	1.16–65.10	0.03	6.83	1.11–41.98	0.038
Recent surgery	0.01	0.00–7.06	0.18	0.00	-	0.99	0.00	-	0.99
Cancer	79.87	0.93-68,831.41	0.20	-	-	-	0.00	-	0.99
Immunosuppression	0.91	0.04–21.21	0.95	-	-	-	5.09	0.81–31.89	0.05
Hospitalization length							0.51	0.09–2.81	0.44
Lab tests									
WBC > 15.000	0.99	0.99–1.00	0.20	1.00	0.99–1.00	0.55	0.99	0.99–1.00	0.26
CRP > 5 mg/dL	1.00	0.99–1.02	0.18	1.00	0.99–1.01	0.28	0.99	0.96–1.01	0.56
PCT > 1 ng/mL	0.81	0.45–1.46	0.50	0.95	0.50–1.80	0.89	0.57	0.24–1.34	0.20
Albumin < 3 g/L	0.32	0.43–1.49	0.28	0.18	0.13–2.60	0.21	0.37	0.01–8.38	0.53
PCR findings									
*Campylobacter* spp.	7.48	0.77–72.31	0.04						
*C. difficile*	13.37	1.08–165.1	0.004						

## Data Availability

Data are available based on request from the corresponding author.
